# A Performance Analysis of Public Expenditure on Maternal Health in Mexico

**DOI:** 10.1371/journal.pone.0152635

**Published:** 2016-04-04

**Authors:** Edson Servan-Mori, Leticia Avila-Burgos, Gustavo Nigenda, Rafael Lozano

**Affiliations:** 1 National Institute of Public Health, Cuernavaca, Morelos, Mexico; 2 School of Medicine, State of Morelos Autonomous University, Cuernavaca, Morelos, Mexico; 3 Institute for Health Metrics and Evaluation, UW, Seattle, WA, United States of America; National Institute of Health, ITALY

## Abstract

We explore the relationship between public expenditure, coverage of adequate ANC (including timing, frequent and content), and the maternal mortality ratio -adjusted by coverage of adequate ANC- observed in Mexico in 2012 at the State level. Additionally, we examine the inequalities and concentration of public expenditure between populations with and without Social Security. Results suggest that in the 2003–2011 period, the public expenditure gap between women with and without Social Security decreased 74%, however, the distribution is less equitable among women without Social Security, across the States. Despite high levels of coverage on each dimension of ANC explored, coverage of adequate ANC was lower among Social Security than non-Social Security women. This variability results in differences up to 1.5 times in State-adjusted maternal mortality rate at the same level of expense and maternal mortality rate, respectively. The increase in the economic resources is only a necessary condition for achieving improved health outcomes. Providing adequate health services and achieving efficient, effective and transparent use of resources in health, are critical elements for health systems performance. The attainment of universal effective coverage of maternal health and reducing maternal mortality in Mexico, requires the adjustment of policy innovations including the rules of allocation and execution of health resources. Health policies should be designed on a more holistic view promoting a balance between accessibility, effective implementation and rigorous stewardship.

## 1. Introduction

Allocation of public funding on health care based on demographic and non-demographic determinants (technological and institutional progress and the market structure) is a first-order task for most governments around the world. In general terms, public spending on health seeks to impact on general health conditions through two ways, first by improving the accessibility to health services through increased availability of productive inputs (capital, work force and technology); and, second, relaxing the budget constraint of consumers and influencing their decisions and consumption patterns of health. This means that theoretically higher spending and investment in health in any health care system, should be associated with better health outcomes. However, the increase in the financial resources is only a necessary condition for achieving improved health outcomes. Ensuring adequate health services, acceptable levels of financial protection, a fair distribution and efficient use of health resources, are critical elements in health systems to achieve effective coverage [1,2], and improve population’s health status.

One of the health systems outcomes, maternal mortality, can illustrate the problems in resource allocation. Its importance lies in its link with inequality, the social and economic backwardness of most developing countries, including Mexico. Additionally, a large proportion of maternal mortality is theoretically avoidable, and it reflects the presence of serious manifestations of inequity in the access to health services [3–10]. Recent estimations suggests that, out-off total maternal death estimated for 2013 (292,982 women, 99.4% in developing countries), 52.3% occurred antepartum, intrapartum and postpartum [11]. Although various criticisms have emerged on the appropriate definition and utilization of MDGs indicators, most countries are using them to monitor their achievements in maternal and infant mortality. In Mexico, maternal mortality ratio has declined around 48.4% during 2000–2013 but according to the Ministry of Health [12], this reduction is insufficient to achieve the MDG commitment of a reduction of 75% to 2015 (equivalent to 22.2) [13]. Most of these deaths are related to the lack of skilled health personnel in basic or emergency obstetric care level [8], and factors related to timely detection and effective obstetric treatment [14].

The Mexican health care system is population-segmented according to employment in the formal sector of economy, the informal services production and the agricultural production [15]. Formal sector employees are enrolled by the Social Security sub-system, being the most important institution the Mexican Institute of Social Security (IMSS in Spanish), which provides services to private sector workers. According to the 2012 National Health and Nutrition Survey (ENSANUT in Spanish), IMSS enrollments represented 30.4% of the total population of 115 million. IMSS is followed by the Social Security Institute for Government Employees (ISSSTE in Spanish), which provides health services to federal government and state employees, covering 5.6% of the population. Social Security provides a broad variety of prepaid interventions, services, and medicines included in the institutional formulary. The Ministry of Health (MoH, the public sub-system) provides care to all individuals not covered by Social Security. *Seguro Popular de Salud* (SPS), introduced in 2003, is a voluntary scheme of public insurance (in-built within the Ministry of Health) for population without access to Social Security. In 2012 ENSANUT reported that over 36% of people were enrolled to Seguro Popular and 25.4% reported having no health insurance at all [16].

In the last fifteen years, the Mexican health system has implemented important health reforms and policies aimed at ensuring equity in health care access [17], and improved health outcomes [18]. Particularly in maternal health, policy has been oriented towards guaranteeing access to family planning and maternal health care and to ensure equity across the health system. The Action Program “*Arranque parejo en la Vida*” (Fair Start of Life) [18] was implemented in 2001 (and halted in 2006) for the public sub-system in regions of Mexico with underdevelopment and high mortality rates in order to strengthen family planning services and health care of child delivery by qualified personnel. While this program succeeded in increasing the number of antenatal consultations among women without social security, it was insufficient for improving child delivery health services, precisely in the indigenous rural areas [19]. The establishment (in 2003) of the System of Social Protection in Health, and its financial branch, the SPS, provided a mechanism for financial protection for the population without social security, and seeked to achieve universal, effective and timely access to a package of health services without co-payment at the point of care [20,21]. Since the beginning of its formal launch the total public budget designated to operate SPS has grown 1,955% starting from 687 to 11,979 million dollars between 2004 and 2013 [22]. During 2008 the strategy Healthy Pregnancy was implemented at the national level with the objective of providing all pregnant women, lacking access to social security, with financial protection through their affiliation to the SPS, which guaranteed free antenatal, delivery and puerperium care. In the same year, the Interinstitutional Agreement for Obstetric Emergency Care was signed among Social Security institutions and the Ministry of Health with the commitment to care all women with an obstetric emergency without the health insurance scheme; no institution can turn away a woman when facing an obstetric emergency on the ground of lacking insurance [18]. Over this period, deliveries by physicians increased by 8% betweeen 2000 and 2012 [23,24], 1.8 million of pregnant women adscribed to SPS [12], and the number of antenatal visits per pregnant women increased from 4.4 to 5.5 between 2008 and 2012 [23]. Family planning coverage on contraceptive methods increased by 5% during 1997–2012 [12,23].

Despite the scaling-up in financial resources, the achievements in financial protection, the increase of health care coverage attributable to the process of reform of the Mexican Health Care System, and the growth in the level of spending on health observed in Mexico in the last decade (24.7% of GDP in the period 2003–2012), the volume of public resources for health care are still lower than those observed in other countries with similar development, including the Latin American region. For example, public spending on health in Mexico in 2012 was equivalent to 41% of the spending observed among the OECD countries [25]. Furthermore, the benefits have been distributed in a heterogeneous manner among social groups (particularly among adolescents, without Social Security, indigenous groups and residents from rural areas), presenting serious challenges to the Mexican State’s health systems in terms of coverage and access to services [26–29].

Most of the growth of public financing in the system has been attributed to SPS. As mentioned above, SPS aims to improve the public sub-system’s performance and to redress the historical financial gaps existing within the sub-system, and between the public (Ministry of Health) and Social Security sub-systems. Unlike Social Security, the public sub-system is decentralized in 32 federal entities, each one of which receives funds from a central agency to guarantee the provision of a package of 284 primary and secondary interventions [2]. Innovations also included a new rationale for the allocation of resources aiming to break historical budgeting and introducing per capita financing in order to stimulate responsiveness of local systems to population needs. Implementation evaluations made to Seguro Popular, however, have shown a great heterogeneity in the way resources are used and the level of achievements obtained [30]. The major challenges identified in these evaluations was the following: lack of coordination between Seguro Popular central agency and local managerial entities, lack of managerial capacity and experience at local level, weak implementation and use of information systems, and interference of political interests in the technical definition of priorities [31]. All these issues tend to reduce the capacity of local systems and Seguro Popular as a whole, to make the most efficient and effective use of financial resources for the benefit of its enrolled population [32].

Based on these elements, our study seeks to move beyond the sole allocation of financing to answer the following questions: Which is the magnitude of the reduction in public investment between public (MoH) and social security sub-systems? What are the differences in terms of antenatal care coverage and maternal health among states? What is the relationship between these differences and health expenditure? Our study explores the existence of relationship between the accumulated expenditure in maternal health services at the State level, the timing, frequency and content of ANC (or adequate ANC), and the maternal mortality ratio -adjusted by coverage of adequate ANC- observed in Mexico in 2012. Additionally, we examine the inequality and concentration of the public expenditure on maternal health through the estimation of the Gini index and Lorenz curve among population with or without Social Security.

## 2. Methods

### 2.1 Settings

The units of analysis were Mexico’s 31 Federal States and 1 Federal District. We explored three main variables. First, the public expenditure (at constant prices of 2011, US$) accumulated between 2003 and 2011 at State level in maternal health services (antenatal care, childbirth and puerperium) [33]. These data was obtained from the Reproductive Health Subaccounts [33] (available in https://dx.doi.org/10.6084/m9.figshare.3121678.v1), and are based on the System of Health Accounts of the WHO/World Bank/USAID [25], and allow analyzing the public and private financial resources of the Mexican health system executed in programs and actions towards reproductive health. Public expenditure was estimated at State level by type of health insurance scheme (Social Security and Ministry of Health).

Second, adequate ANC coverage was defined using the WHO criteria [34] and information about maternal health of approximately 9.1 million women aged 12–49 who had their last childbirth during 2006–2012, and were participants in the 2012 ENSANUT [16]. The data from ENSANUT were requested and obtained from the surveys public repository hosted at the National Institute of Public Health (NIPH). The Institutional Board (Ethics Committee) at the NIPH in Mexico reviewed and approved the survey. All interviewees provided verbal informed consent prior to participating (See details in http://ensanut.insp.mx/basesdoctos.php#.UvvEGrSK98F).

According to other studies [35], adequate ANC care was defined as having the first medical visit within the first trimester of pregnancy, ≥4 ANC visits, and ANC included ≥75% of recommend care (out of 11 basic care procedures: measurement of height, weight, and blood pressure, urinalysis, blood examination, blood sugar examination, syphilis detection-VDRL-, ultrasound, tetanus vaccine, folic acid, vitamins/iron/food supplement). Following our previous work, we assumed that each of the care procedures is equally important [29].

Third, we analyze data published by the Mexican government about maternal mortality ratio at the State level in 2012 [36] adjusted by coverage of adequate care. This variable was based on the WHO Guidelines for the implementation of the International Classification of Diseases to the deaths during pregnancy, childbirth and postpartum [37], and includes two types of maternal deaths: First, deaths during pregnancy or within 42 days following the end of the pregnancy, regardless of the length and location of the pregnancy, due to any cause related to or aggravated by pregnancy or its management but not from accidental or incidental causes; and, Second, deaths occurred from direct or indirect causes more than 42 days but less than one year after termination of pregnancy (or late maternal deaths). Both in a given year, per 100 thousand live births in that same year and per percentage point of adequate ANC coverage. The total number of births were obtained from official estimates by the National Population Council of Mexico [38].

### 2.2 Analysis

We describe the evolution (at State level) of the public expenditure on maternal health during 2003–2011 expressed in US$ and per women in reproductive age (15–49 yrs) [39] with or without Social Security.

Next, we describe socio-demographic and retrospective ANC characteristics of women participants reported by the 2012 ENSANUT by type of insurance. Women without Social Security includes those without any type of health insurance and those who reported being affiliated to the SPS. We used bivariate regression models accounting for the complex survey design to compare the groups and permit population-level estimates. These characteristics include:

1. Schooling (yrs); an asset index as proxy of socio-economic level using principal components analysis with polychoric correlation matrices [40], based on household-level assets and infrastructure [range: -5.89;1.83]; more positive scores indicate higher socio-economic condition, while lower socio-economic condition households have more negative scores; an indicator for being a part of a beneficiary household of the *Oportunidades* program (currently *Prospera*).
2. Characteristics at the time of the most recent child birth as: age (yrs), number of children (0, 1, and ≥2), history of stillborn child or a child died before the first year of life, and abortion or miscarriage, a proxy of pregnancy risk that include (in a binary variable 1/0) the occurrence of high blood pressure, vaginal bleeding, anemia, threat of miscarriage, preeclampsia or eclampsia, gestational diabetes, or infections during pregnancy, the frequent care provider (Social Security, Ministry of Health, private and midwife/home, or other).
3. Related to residence area and following or using the ENSANUT criteria: Rural neighborhoods were defined as locations with less than 2,500 inhabitants, urban areas were those with 2,500 to 100,000 inhabitants, and metropolitan locations were areas with greater than 100,000 residents, and the degree of marginalization (low/high, based on access to basic infrastructure services, housing conditions, education attainment, and wage earnings) [41].

Combining information from cumulative public expenditure between 2003 and 2011 on maternal health services and estimation of adequate ANC coverage at State level, we analyzed the equality and concentration of the public expenditure through the estimation of the Gini index [42] and Lorenz curve [43] between population with or without Social Security. These adequate ANC coverages were estimated using non-linear regression models (logit) adjusted by all population and contextual characteristics mentioned above, accounting for the complex survey design. This strategy allowed us to control the demand and contextual characteristics, in order that the observed variation in the levels of estimated coverages would be explained, in part, by the performance of the State health systems. The F-adjusted test statistic = 0.692 (Prob>F = 0.717) and the specification link-test suggest that the model estimated had an acceptable goodness-of-fit and was correctly specified (variable of prediction was statistically significant, and squared predictor was not significant) [44].

Finally, in order to analyze the State health systems performance in terms of public expenditure on maternal health and maternal results (input/output), we explored the relationship between the cumulative public expenditure between 2003 and 2011 and the maternal mortality ratio adjusted by coverage of adequate ANC at state level. We used Stata 13.1 for all analyses.

## 3. Results

**Fig 1:**
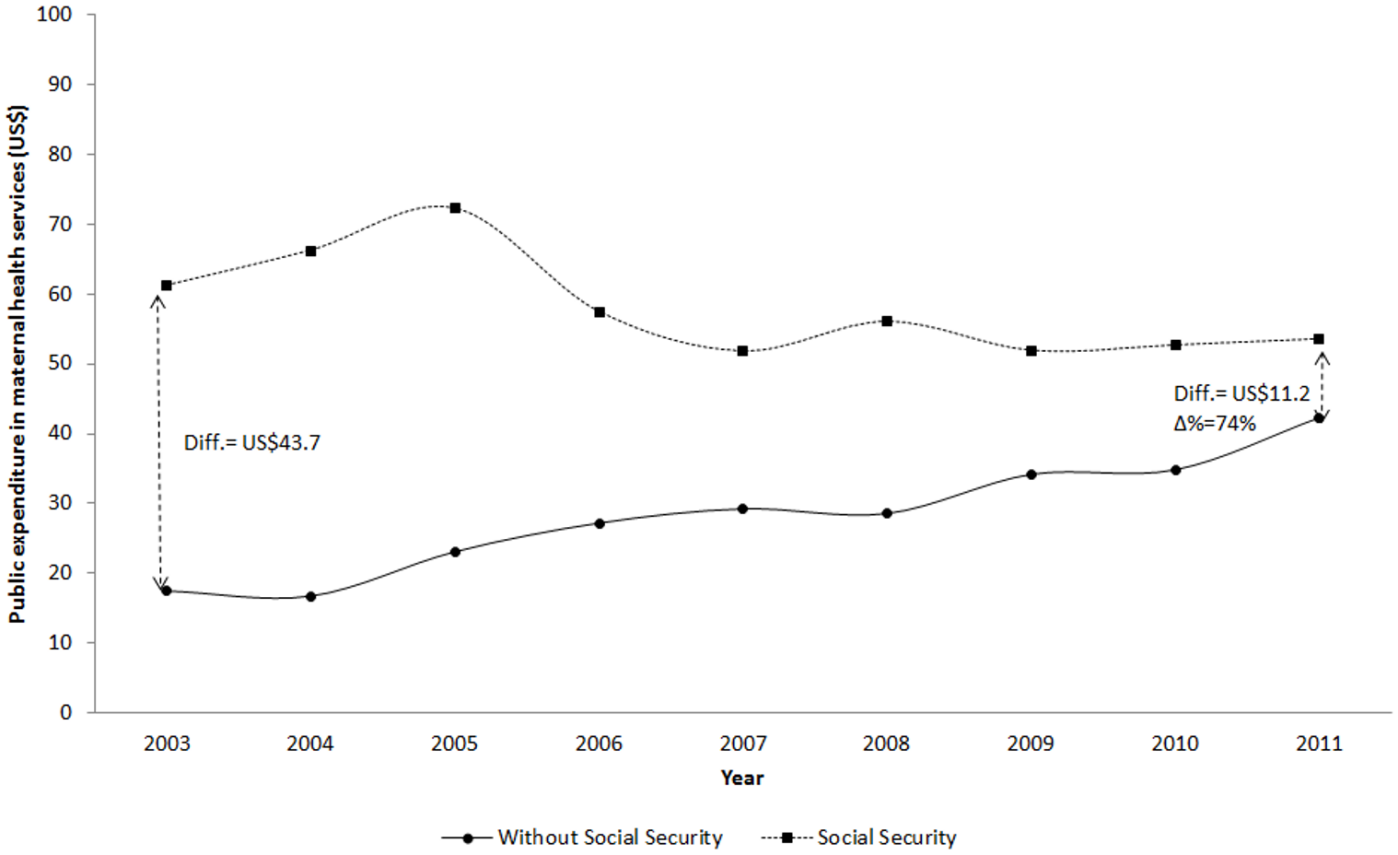
Public expenditure on maternal health services per women -in reproductive age- with or without Social Security. Note: * Expressed in US$ and per women 15–49 years of age (at constant prices of 2011). **Includes women without any type of health insurance and those who reported being affiliated to the *Seguro Popular de Salud* (SPS).

According to data of public expenditure on maternal health, in 2003 the gap between population with and without Social Security per women 15–49 years was more than 40US$, favorable to the first. In 2006, the magnitude of this gap was reduced to 43.7US$; after 8 years of observation, in 2011 this difference was 11.2US$, which represented a 74% reduction in the gap (Fig 1).

Tables 1 and 2 present the socio-demographic and ANC services received by insurance affiliation. Women without Social Security reported higher parity (38% reported ≥2 children vs 29.9% among women with Social Security), a greater percentage of at least one stillborn child or a child who died before the first year of life (4.6% vs 2.5% in women with Social Security). Among women without Social Security, 59.9%, received ANC at any Ministry of Health (59.9%), Social Security (14.5%) or private (22.7%) facility (vs 12%, 63%, and 23.6% among Social Security women) respectively. Women without Social Security had fewer schooling, are more likely to reside in indigenous, poor, and *Oportunidades*-beneficiary households, and rural and isolated areas. Tables also shows that nearly all women receive ANC (99.4% with Social Security vs 98.0% without Social Security). Women without Social Security report lower coverage of timely ANC or first consultation during the first trimester of pregnancy (80.4% vs 89.8%), received ≥4 ANC visits (89.4 vs 96.0%), and the report of ≥75% of the basic procedures during ANC (83.5% vs 88.0%). Despite high levels of coverage on each dimension of ANC explored, coverage of adequate ANC (the combined indicator previously mentioned) was 63.2% [CI95%:61.0;65.5] among women without Social Security and 76.4% [CI95%:72.9;80.0] among women with Social Security.

**Table 1:** Socio-demographics characteristics among women -in reproductive age- with or without Social Security.

	Without Social Security*	With Social Security
Weighted Sample	6,346,877	2,720,090
%	70.0	30.0
	Average and percentage [CI95%]	
**Socio-demographic characteristics**		
Age (yrs.)	24.8 [24.6;25.1]	26.6 [26.2;27.1]^§^
Number of children		
0	32.0 [29.7;34.3]	33.9 [30.4;37.4]
1	30.0 [27.9;32.2]	36.2 [32.8;39.5]^§^
≥2	38.0 [35.7;40.2]	29.9 [26.8;33.0]^§^
Child dead at childbirth or during the 1st yr.	4.6 [3.7;5.4]	2.5 [1.4;3.5]^§^
At least one miscarriage or abortion	13.8 [12.3;15.2]	16.6 [14.3;19.0]^§^
Diagnosis of some health problem	56.0 [53.5;58.4]	62.8 [59.3;66.3]^§^
Frequent care provider		
Social Security	14.5 [12.6;16.4]	63.0 [59.2;66.7]^§^
Ministry of Health	59.9 [57.3;62.5]	12.0 [9.6;14.3]^§^
Private	22.7 [20.4;25.0]	23.6 [20.3;26.9]
Other	2.9 [2.3;3.6]	1.5 [0.7;2.3]^§^
**Other individual characteristics**		
Schooling (yrs.)		
Zero	5.3 [4.3;6.4]	2.2 [1.1;3.4]^§^
1–6	27.4 [25.3;29.6]	8.8 [6.9;10.6]^§^
7–9	39.8 [37.5;42.2]	33.7 [30.0;37.4]^§^
10–12	20.5 [18.5;22.5]	33.8 [30.1;37.5]^§^
≥13	6.9 [5.6;8.3]	21.5 [18.6;24.3]^§^
Indigenous	12.0 [10.3;13.8]	3.9 [2.7;5.0]^§^
Oportunidades beneficiary	29.1 [27.0;31.2]	8.2 [6.5;9.9]^§^
Asset index (SES proxy, in quintiles)		
I	26.5 [24.3;28.7]	4.9 [3.7;6.1]^§^
II	22.9 [21.1;24.7]	13.4 [11.3;15.5]^§^
III	21.0 [19.0;23.1]	22.4 [19.8;25.1]
IV	16.6 [14.5;18.6]	24.5 [21.2;27.7]^§^
V	13.1 [11.2;15.0]	34.8 [31.0;38.7]^§^
Rural	28.6 [26.8;30.4]	10.3 [8.1;12.5]^§^
Urban	22.3 [20.7;24.0]	15.3 [13.7;16.9]^§^
Metropolitan	49.1 [46.7;51.4]	74.4 [71.8;77.0]^§^
Low marginalization	70.4 [68.4;72.4]	88.6 [87.0;90.1]^§^
High marginalization	29.6 [27.6;31.6]	11.4 [9.9;13.0]^§^

Note: Estimations based on the effect of the survey design.

*Includes women without any type of health insurance and those who reported being affiliated to the Seguro Popular.

^§^Difference at 1 or 5% level.

**Table 2:** Antennal care characteristics among women -in reproductive age- with or without Social Security.

	Without Social Security*	With Social Security
Weighted Sample	6,346,877	2,720,090
%	70.0	30.0
	Average and percentage [CI95%]	
**Prenatal period: Access to and quality of care**		
Access to Antenatal Care (ANC)	98.0 [97.5;98.5]	99.4 [98.9;99.8]^§^
Timely (1^st^ ANC visit during the first quarter (A)	80.4 [78.7;82.1]	89.8 [87.4;92.2]^§^
Frequent (At least four consultations) (B)	89.4 [88.0;90.8]	96.0 [94.7;97.3]^§^
Content of ANC consultation (C)	83.5 [82.5;84.4]	88.0 [87.1;88.9]^§^
Adequate ANC: (A)+(B)+[(C)≥75%]	63.2 [61.0;65.5]	76.4 [72.9;80.0]^§^

Note: Estimations based on the effect of the survey design.

*Includes women without any type of health insurance and those who reported being affiliated to the Seguro Popular.

^§^Difference at 1 or 5% level.

**Fig 2:**
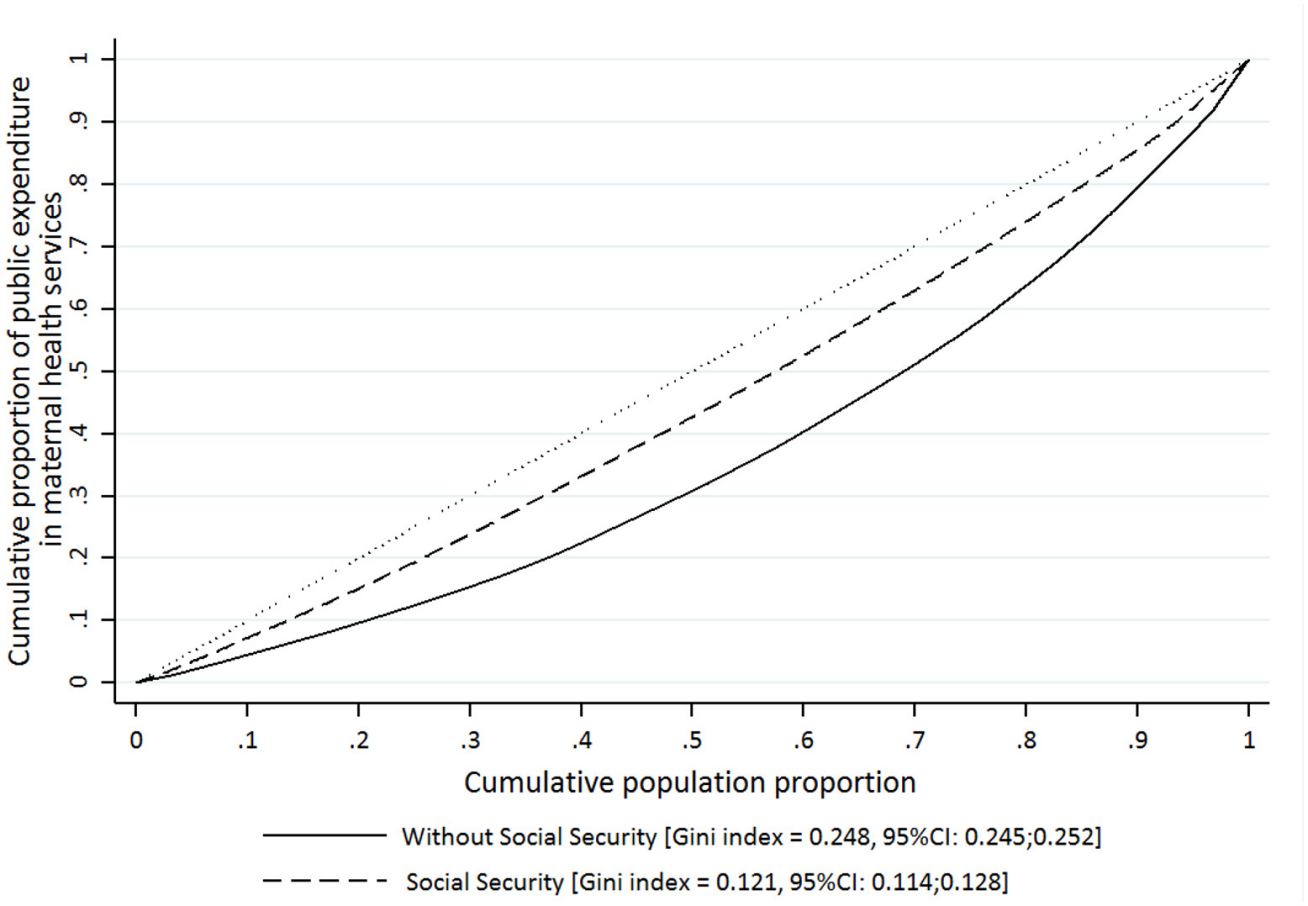
Lorenz curve and Gini index related to the public expenditure in maternal health services among women -in reproductive age- with or without Social Security. Note: Expressed in US$ and per women 15–49 years of age (at constant prices of 2011). **Includes women without any type of health insurance and those who reported being affiliated to the Seguro Popular.

**Fig 3:**
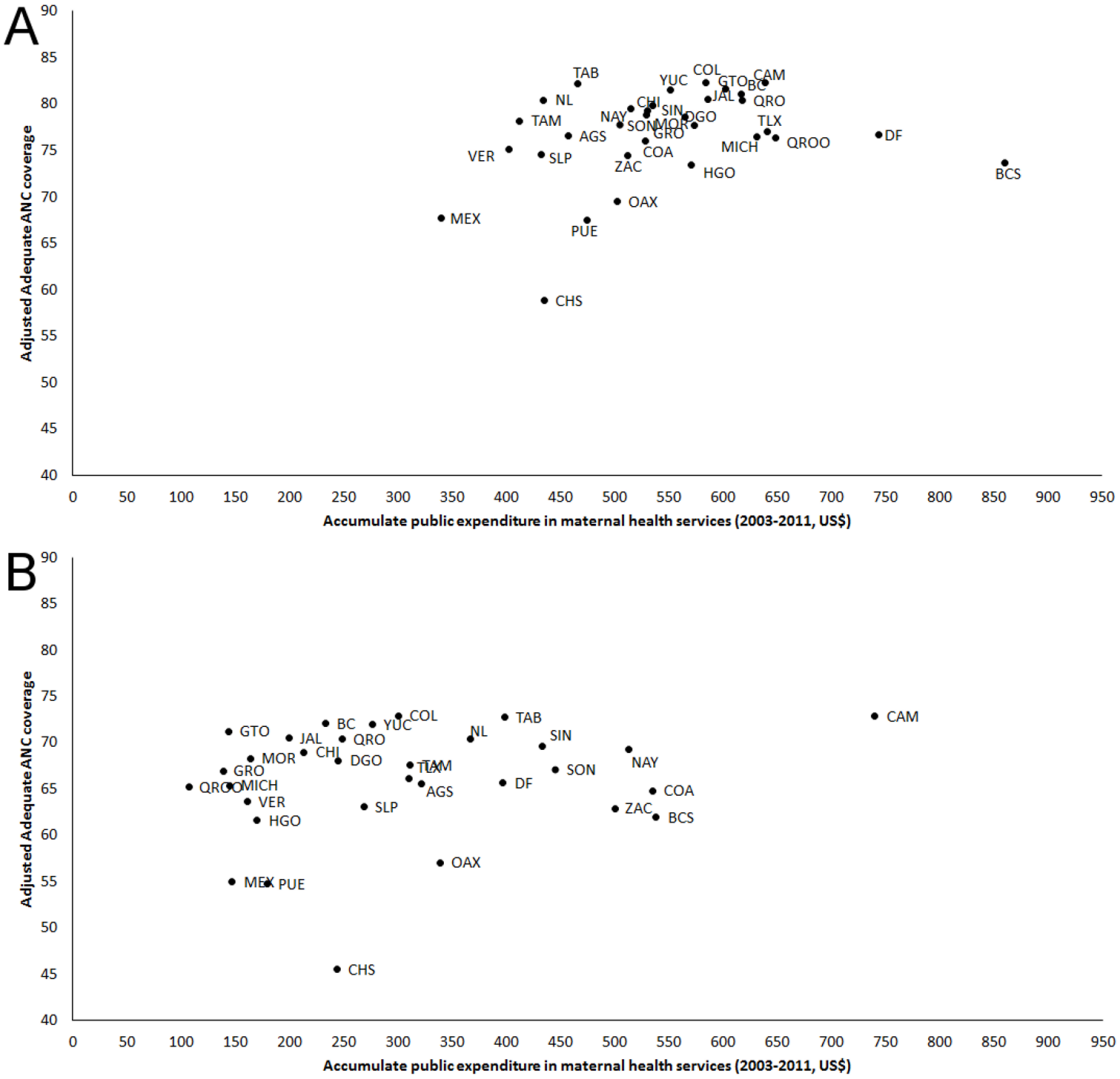
Adequate antenatal care coverage and public expenditure in maternal health services among women -in reproductive age- with or without Social Security. **PANEL A.** Social Security. **PANEL B.** Without Social Security*. Note: *Includes women without any type of health insurance and those who reported being affiliated to the Seguro Popular. **Adequate ANC (timely, ≥4 ANC visits + content of ANC ≥75% of basic procedures) were estimated using non-linear regression models (logit) adjusted by all population and contextual characteristics mentioned in Table 1. ***Expressed in US$ and per women 15–49 years of age (at constant prices of 2011).

Fig 2 shows the cumulative public expenditure on maternal health 2003–2011 in the Mexican population according to the health insurance scheme. We observed that Social Security women presented a more concentrated distribution of public expenditure [Gini index = 0.104; CI95%:0.10;0.11] compared to women without Social Security [Gini index = 0.264; CI95%:0.26;0.27]. There are two possible sources of public expenditure concentration in the case of women without Social Security (Fig 3): (1) There are differences at the coverage level of adequate ANC at similar levels of public expenditure (i.e. Chiapas-CHS- were the estimated expenditure was 243.9US$ with a 45.5% coverage, compared to Baja California-BC- were the level of expenditure was 233.0 US$ with a coverage of 72.1%; this represented a 58.5% higher coverage with an expenditure increase of 4.7%). (2) There are important differences in the levels of public expenditure at similar coverage level (i.e. Campeche-CAM- where the estimated level of expenditure was 739.7US$ and coverage of 72.9%, compared to Guanajuato-GTO- where this estimates were 143.7US$ and 71.2% respectively).

**Fig 4:**
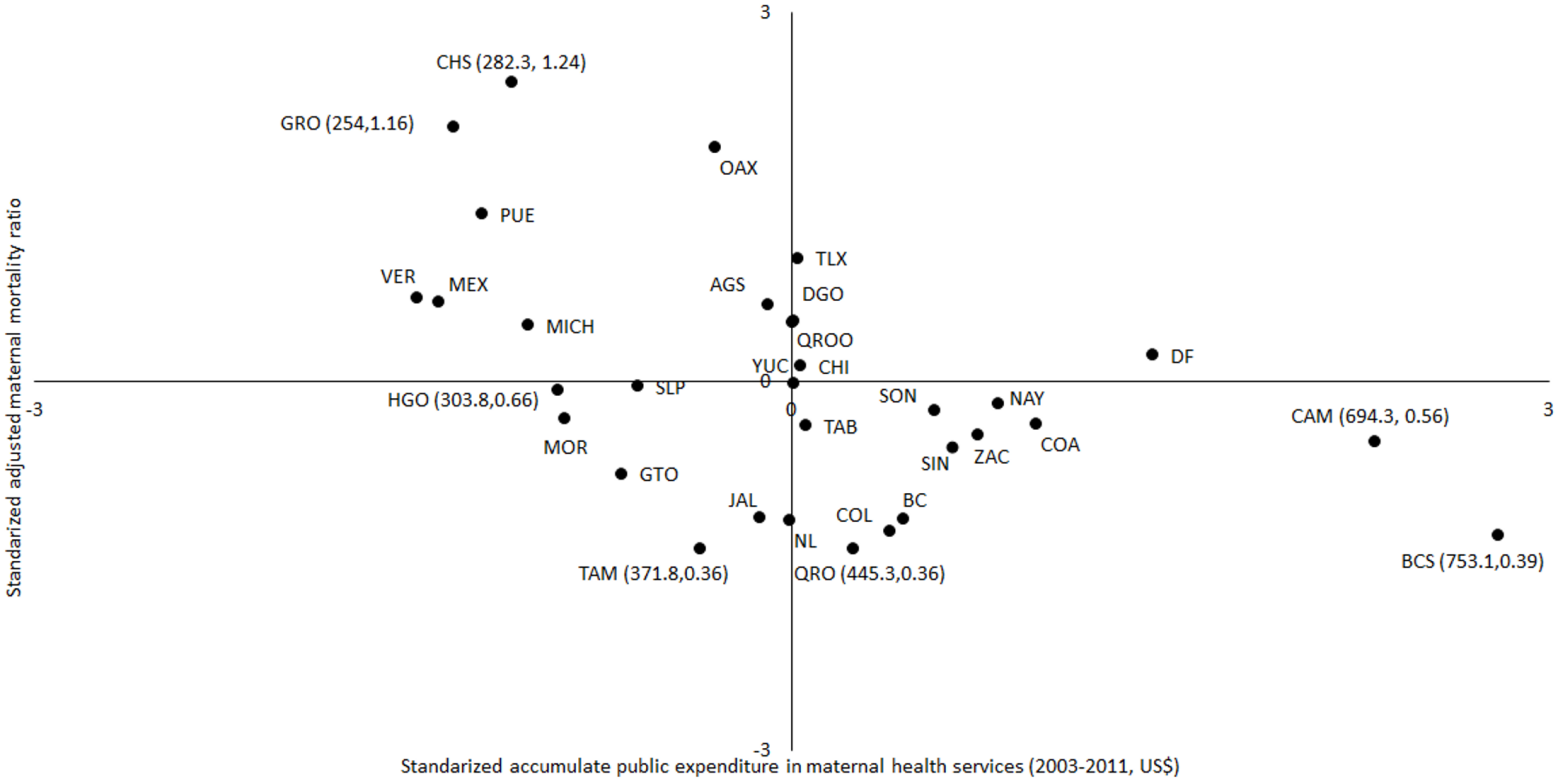
Performance of Mexican states in Maternal Health: Public expenditure and maternal mortality/antenatal care coverage results. Note: In parenthesis absolute values. Maternal mortality was based on the WHO Guidelines for the implementation of the International Classification of Diseases to the deaths during pregnancy, childbirth and postpartum (37), and includes two types of maternal deaths: First, deaths during pregnancy or within 42 days following the end of the pregnancy, regardless of the length and location of the pregnancy, due to any cause related to or aggravated by pregnancy or its management but not from accidental or incidental causes; and, Second, deaths occurred from direct or indirect causes more than 42 days but less than one year after termination of pregnancy (or late maternal deaths). Both in a given year, per 100 thousand live births in that same year and per percentage point of adequate ANC coverage. *Adequate ANC (timely, ≥4 ANC visits + content of ANC ≥75% of basic procedures) were estimated using non-linear regression models (logit) adjusted by all population and contextual characteristics mentioned in Table 1. **Expressed in US$ and per women 15–49 years of age (at constant prices of 2011).

Fig 4 shows the results of the performance of state health systems in terms of standardized public expenditure on maternal health and the maternal mortality ratio adjusted by adequate ANC coverage. As it can be observed, Chiapas (CHS) presents the lowest performance in terms of inputs (expenditure) and outputs (maternal mortality ratio), i.e. at similar levels of public expenditure on maternal health as Hidalgo (HGO) [approximately 200US$], Chiapas presented 1.5 times higher maternal mortality. Interesting to highlight is that BCS, when compared to Queretaro (QRO), had almost twofold of public expenditure at similar maternal mortality level.

## 4. Discussion and Conclusion

Mexico has been reforming its health care system for the last 30 years. The new phase of reform and policies started in 2003 with the implementation of *Seguro Popular de Salud* or SPS raising enormous expectations about its achievements [45,46]. In the 1990s a wave of reforms expanded over a broad range of developing countries and in Latin America a major feature was the increase of public expenditure [47]. Furthermore, countries expanding public expenditure decided to incorporate managerial innovations to allocate funds through different modalities. A great interest by analysts has been focused on which allocation modality accounts for best results. Unlike other countries in the region such as Colombia or Chile, Mexico has concentrated its reforms and policies in the public sub-system aiming at reducing financial gaps and increasing access of non-insured populations to a broad range of services. The account of the Mexican reform’s broader systemic effects should be the subject of further studies. In this paper we focus on a key and sensitive aspect of health care provision, maternal care which is one of the determinants of maternal mortality, and its relationship with public health expenditure in maternal health services at State level among population with or without Social Security.

In other developing countries, it has been demonstrated that besides increasing public expenditure, health systems should re-organize the provision of health services in order to achieve health results. For example, for the case of maternal mortality [48], it has been shown that the main factors behind the success of Chile to reduce maternal and infant mortality to the lowest regional level is related to the longstanding provision of quality individual and community health services in urban and rural areas. Reforms have shown weaknesses in strengthening community health services, as their strategies tend to focus on the provision of personal services [49].

Our results show that despite the reduction of the gap of public expenditure between Social Security enrolled and not enrolled populations [2], differences in the level of provision, the quality of care and the effects on populations’ health are still striking. Results show that the level of investment and provision at Social Security institutions is more homogeneous than the Ministry of Health. Social Security institutions are centralized hierarchical structures that define standards of care and investments that apply in the whole country and variations are clearly reduced. On the opposite, the Ministry of Health is a decentralized structure where historical differences between rich and poor states go beyond population characteristics and permeate also the organization of local Ministries of Health and their performance. SPS financing was anchored in this structure and while partially achieving its financial redistribution goals is finding problems to achieve operational and performance goals [32].

In terms of antenatal care (ANC), even though coverage was high in both women (with or without Social Security), when combining timeliness (the first medical visit within the first trimester of pregnancy), frequency (or four ANC visits) and content of ANC (≥75% of recommend care process), only two out of three women without Social Security and three out of four women with Social Security received adequate ANC. Since both conditions (timely ANC as well as four ANC visits) are relevant to achieve reduction in ANC achieving higher adequate coverage is necessary in both groups but particularly in women without Social Security. Other studies suggest problems of accessibility to health care units [27].

Most of accessibility restrictions mentioned above, particularly affecting populations served by the Ministry of Health, are in part the expression of health services organizational and management flaws. For example, insufficient supply of inputs, such as medicines, are more severe in Ministry of Health than in Social Security services. This could contribute to explain the lower proportion of adequate ANC observed among women without Social Security. The map of the distribution between public investment and high quality of health services provision (Fig 3) and the map between public investment and maternal mortality (Fig 4) show two adjacent and complementary phases of this problem. After controlling for the main characteristics of demand (shown in Table 1), while the first one shows that not all states may be using resources appropriately to produce quality services, the second shows that this investment would not produce health improvements if quality is not increased. Certainly, part of this heterogeneity may be due to differences in structural conditions (poverty, infrastructure, etc.) and not to public expenditure; however, a large part may be explained by differences in the technical and provision services performance and the criteria applied for allocation of financial resources among States. On the one hand, as mentioned above, while for Social Security, decisions on resource allocation and purchasing inputs are performed centrally, in the case of the population without Social Security, each State Ministry of Health assume the responsibility for the amount and type of personnel to be hired and the purchasing of inputs [32,50]. External evaluations of SPS implementation in states suggest a lack of both independence and separation of functions between the SPS state financial intermediaries (financiers) and State Ministry of Health (service providers), redounding in deficient control, supervision and accountability of resources [32,51]. This, coupled with larger transfers of resources and fragmented funding sources, could lead interference of political interests in the definition of priorities within state ministries with weak management and control systems, as well as deviation of resources. Future studies will need to analyze these aspects, which have been shown as relevant to spending efficiency [52,53].

We also found a greater inequity in the distribution between both women groups (with or without Social Security, Gini index = 0.10 vs. 0.26, respectively) among States. This is explained by the manner in which public sub-system (Ministry of Health) federal resources have been historically allocated among states. In 2000, spending differences reached 6:1 between states with higher/lower resources [2,51]. The introduction of capitated payments in transferring additional SPS resources [45] and strategies for improving access to maternal health care services for the population without social security have improved allocation among states; however, differences—albeit narrower- clearly persist. One reason for this may be that the State Ministries with fewer resources and lower levels of commitment have reduced their participation in health financing and become more dependent on federal resources [32,51]. Another reason could be the persistence of historical gaps in the provision of infrastructure and resources for the public sub-system, and the existence of differential capacities in the collection and management of financial resources at the state level [30]. Future research should specifically explore these differences and the real weight of resources in the distribution processes.

Our research had several limitations: First, despite the high quality information collected in the 2012 ENSANUT, this is a cross-sectional and self-reported dataset, so causal inference is limited and exist a potential information bias due to the temporality of the ANC indicators (recall bias). Additionally, ENSANUT does not collect information about maternal mortality. Second, there is a lack of standardization in the processes of collecting and reporting financial information on state health systems, as well as a huge gap between the use and report of financial resources, particularly in schemes that provide care to people without Social Security, which could introduce errors of comparability and evaluation of public financing schemes. A further limitation is the loss of registration of maternal deaths due to the way official data is collected and organized. There is a certain level of delay in the report of deaths in the records. It has been estimated that around 12% of maternal deaths are not included in the official reports due to delays in the flow of information. Two more reasons of loss are that maternal deaths of migrant women are frequently difficult to register, and that there is a fraction of the population that tend to move through different insurance schemes or demand private services where is more difficult to do the registration of maternal deaths.

In conclusion, this study shows that the increase in the economic resources is only a necessary condition for achieving improved health outcomes. Providing adequate health services and the efficient, effective and transparent use of resources in health, are the critical elements in health systems performance [54]. Achieving universal effective coverage of maternal health and reducing maternal mortality in Mexico, requires the adjustment of policy innovations including the rules of allocation and execution of resources for health. Health policies should be designed on a more holistic view, including comprehensive knowledge of the socio-demographic profile, health and economic development of the target population, looking for a balance between accessibility and quality via effective implementation and rigorous stewardship. Future studies may be oriented towards the analysis of the relative weight of maternal health expenditure on the use of services and maternal mortality, analysis on the resource allocation efficiency, allocation rules and compliance of the allocation, and the role of health personnel in the production of adequate services. It is necessary to produce periodic, standardized, and more disaggregated information at locality level, and not only at the State level, of total public expenditure and other items (including maternal), as well as, the inputs required for health services production.
